# Hepcidin Status in Cord Blood: Observational Data from a Tertiary Institution in Belgium

**DOI:** 10.3390/nu15030546

**Published:** 2023-01-20

**Authors:** Michael Ceulemans, Joline Van de Vel, Dorine W. Swinkels, Coby M. M. Laarakkers, Jaak Billen, Kristel Van Calsteren, Karel Allegaert

**Affiliations:** 1 Clinical Pharmacology and Pharmacotherapy, Department of Pharmaceutical and Pharmacological Sciences, KU Leuven, 3000 Leuven, Belgium; 2 Teratology Information Service, Netherlands Pharmacovigilance Centre Lareb, 5237 MH Hertogenbosch, The Netherlands; 3 L-C&Y, Child and Youth Institute, KU Leuven, 3000 Leuven, Belgium; 4 Faculty of Pharmaceutical Sciences, KU Leuven, 3000 Leuven, Belgium; 5 Translational Metabolic Laboratory (TML 830), Department of Laboratory Medicine, Radboud University Medical Center, 6500 HB Nijmegen, The Netherlands; 6 Hepcidinanalysis.com, 6525 GA Nijmegen, The Netherlands; 7 Sanquin Blood Bank, Plesmanlaan 125, 1066 CX Amsterdam, The Netherlands; 8 Department of Laboratory Medicine, University Hospitals Leuven Gasthuisberg, 3000 Leuven, Belgium; 9 Department of Obstetrics & Gynecology, University Hospitals Leuven Gasthuisberg, 3000 Leuven, Belgium; 10 Woman and Child, Department of Development and Regeneration, KU Leuven, 3000 Leuven, Belgium; 11 Department of Hospital Pharmacy, Erasmus MC, 3000 CA Rotterdam, The Netherlands

**Keywords:** pregnancy, cord blood, iron status, iron regulation, hepcidins

## Abstract

The hormone hepcidin plays an important role in intestinal iron absorption and cellular release. Cord blood hepcidin values reflect fetal hepcidin status, at least at the time of delivery, but are not available for the Belgian population. Therefore, we aimed (1) to provide the first data on cord blood hepcidin levels in a Belgian cohort and (2) to determine variables associated with cord blood hepcidin concentrations. A cross-sectional, observational study was performed at the University Hospital Leuven, Belgium. Cord blood samples were analyzed using a combination of weak cation exchange chromatography and time-of-flight mass spectrometry. Descriptive statistics, Spearman correlation tests, and Mann–Whitney U tests were performed. In total, 61 nonhemolyzed cord blood samples were analyzed. The median hepcidin level was 17.6 μg/L (IQR: 18.1; min-max: 3.9–54.7). A moderate correlation was observed between cord blood hepcidin and cord blood ferritin (r = 0.493) and hemoglobin (r = −0.342). Cord blood hepcidin was also associated with mode of delivery (*p* = 0.01), with higher hepcidin levels for vaginal deliveries. Nonetheless, larger studies are needed to provide more evidence on the actual clinical value and benefit of cord blood hepcidin measurements.

## 1. Introduction

Iron requirements are increased during pregnancy as a result of the increased number of red blood cells and circulating blood volume [1]. Iron plays a crucial role in oxygen transport to the fetus. The fetus also stores iron during pregnancy that will be used during its first months of life [2]. Hence, pregnant women are at risk of iron deficiency anemia [2]. This should be avoided at all times, as iron deficiency anemia during pregnancy and early childhood is associated with maternal, perinatal, and neonatal morbidity, including altered cognitive and neurobehavioral outcomes in offspring [2,3,4].

To meet the high iron requirements during pregnancy [5], both iron absorption from the maternal diet as well as cellular iron release within the body should be sufficient. The hepatic 25-amino acid peptide hormone hepcidin hereby plays an important role. Hepcidin inhibits dietary iron absorption in the duodenum and iron release from macrophages and hepatocytes [6]. So, increased hepcidin concentrations result in lower levels of circulating iron. Hepcidin concentrations below or above a specific threshold may be indicative of an increased risk of iron deficiency in women [7]. Although the application of hepcidin is currently mainly limited to research settings, as a regulator of iron homeostasis, hepcidin may be, for example, a useful biomarker of the (oral) bioavailability of iron supplementation in pregnancy, guiding clinicians to appropriately prescribe and monitor iron therapy in pregnant women [5].

Previous studies have shown that maternal hepcidin values decrease in the second and third gestational trimester, probably to meet the increased iron needs due to maternal, fetal, and placental weight gain and growth [8,9,10]. However, in pregnancies complicated with inflammatory conditions such as preeclampsia, maternal hepcidin levels were higher compared to women with uncomplicated pregnancies, potentially limiting the amount of iron available for transplacental transfer [11].

Moreover, cord blood hepcidin values reflect fetal hepcidin status, at least at the time of delivery. Previous studies have found a correlation between cord blood hepcidin levels and cord blood iron status [12,13,14,15,16,17,18,19,20]. Likewise, cord blood hepcidin has been associated with maternal variables (e.g., body mass index or BMI) [13,21], pregnancy outcomes (e.g., gestational age at birth, mode of delivery) [14,15,16], and neonatal outcomes (e.g., birth weight) [17], regardless of some conflicting results across studies. Hence, insight into the relationship between cord blood hepcidin and maternal variables/pregnancy–neonatal outcomes could be instructive to contribute to identifying neonates at high(er) risk of low iron status.

To date, observational data on cord blood hepcidin levels are not available for (pregnant) women living in Belgium. Furthermore, the application of different hepcidin assays with varying calibrators used across previous studies impedes the comparison and utility of hepcidin values, limiting its future research and clinical potential [22,23]. Therefore, by using a validated and internationally accepted hepcidin analysis method [23,24], this study aimed (1) to provide observational data on cord blood hepcidin levels in a Belgian cohort and (2) to determine variables associated with cord blood hepcidin concentrations.

## 2. Materials and Methods

A cross-sectional, observational study was performed at the University Hospital Leuven, Belgium between March and September 2017. Cord blood samples were obtained at delivery from women who participated during pregnancy in the PREVIM study exploring the extent and type of “PREgnancy related use of VItamins and Medication” [25,26]. To be eligible for study participation, women must be at least 18 years old, understand Dutch, French, or English, and have visited the obstetrics department of the university hospital as part of the routine antenatal care during the ongoing pregnancy.

Cord blood samples were collected using serum tubes and subsequently centrifuged and aliquoted at the Department of Laboratory Medicine of the University Hospital. Aliquots of 2 mL were kept between 2–8° degrees for maximum 7 days, followed by storing at −80°. Previously, we have shown that in serum samples, which were kept at 4° for 0–7 days and at −80° for less than 2 years, concentrations of hepcidin-25 remained stable [24].

In December 2017, the aliquots were analyzed in one batch at the “Hepcidinanalysis” lab of the Radboud University Medical Center in the Netherlands. To assess hepcidin-25 concentrations, a validated hepcidin analysis method was used based on a combination of weak cation exchange chromatography and time-of-flight mass spectrometry (WCX-TOF MS) [24]. Peptide spectra were generated on a Microflex LT matrix-enhanced laser desorption/ionization TOF MS platform (Bruker Daltonics). A stable hepcidin-25 + 40 isotope was used as the internal standard for quantification. The lower limit of quantification (LOQ) was 0.5 nM (nmol/L); samples with values below the LOQ were given the value zero. Hepcidin-25 concentrations were provided as nM and converted to μg/L (by multiplying with a factor 2.7894).

In our study, the measurements of cord blood hepcidin (2017) were performed before the assay was further standardized (2019) by using secondary matrix-based reference material that was value-assigned by a primary reference material [27,28]. However, as the standardization only slightly altered serum hepcidin values obtained by the WCX-TOF MS method (i.e., standardized results were a factor 1.054 higher compared to historic results obtained without standardization), the results obtained in our study could be considered as results obtained by a standardized hepcidin assay.

To determine variables associated with cord blood hepcidin, the following variables for which a potential relationship has been shown in earlier studies [12,13,14,15,16,17,18,19,20,21] were assessed: (1) ‘cord blood iron status’ (i.e., hemoglobin, ferritin, serum iron, transferrin and transferrin saturation); (2) ‘pre-gestational BMI’; (3) ‘pregnancy outcomes’ (i.e., onset of labor, mode of delivery); and 4) ‘neonatal outcomes’ (i.e., prematurity (born < 37 weeks), low birth weight (<2500 g), small for gestational age (SGA; <10th birth percentile) [29], and large for gestational age (LGA; > 90th birth percentile) [29]. Customized birth weight centiles were calculated, accounting for gestational age, fetal sex, parity, and single/multiple pregnancies. Data were retrieved from hospital medical records shortly after childbirth.

All data were analyzed using descriptive statistics (i.e., median, interquartile range (IQR), absolute numbers, and percentages). The relationship between cord blood hepcidin and the continuous variables was tested using the Spearman correlation test. A correlation coefficient *r* < 0.3 was considered a weak relationship, between 0.3 and 0.7 a moderate relationship, and > 0.7 a strong relationship. The relationship between cord blood hepcidin and the noncontinuous variables was assessed using Mann–Whitney U tests. The variable onset of labor was dichotomized into ‘spontaneous’ and ‘not spontaneous’ (i.e., induction of labor and none/elective caesarean section). The results were considered significant if *p* < 0.05. Data were analyzed using SPSS Statistics version 28 (IBM Corp, Armonk, NY, USA).

Ethical approval was obtained from the EC Research UZ/KU Leuven (S59516; 25 November 2016). All women provided, while being pregnant, their written informed consent for cord blood sampling at delivery and data collection from hospital medical records.

## 3. Results

In total, cord blood samples were collected from 72 individual women. Median gestational age at birth was 39 weeks (IQR: 1.7). Median pregestational BMI was 22.78 (IQR: 5.91). Two women had a multiple pregnancy. Table 1 provides an overview of the study participants’ baseline maternal and pregnancy-related characteristics as well as pregnancy outcomes, according to cord blood hepcidin levels of the nonhemolyzed samples. With regard to pregnancy complications, the following complications were reported in this cohort: preeclampsia (*N* = 3), gestational diabetes (*N* = 3), placenta previa (*N* = 1), hyperemesis gravidarum (*N* = 1), polyhydramnios (*N* = 1) and cardiac complications (*N* = 1).

In total, 74 neonates were born. Overall, median birth weight was 3290 g (IQR: 478 g) (*N* = 73). The other neonatal outcomes are summarized in Table 2.

In total, 73 cord blood samples were available for hepcidin analysis. One sample did not contain a sufficient amount of blood that was needed for the analysis. In 12 samples, hemolysis had occurred. Only one (hemolyzed) sample had a hepcidin level < LOQ. Overall, the median hepcidin level in the nonhemolyzed (*N* = 61) and hemolyzed (*N* = 12) cord blood samples was 17.6 μg/L (IQR: 18.1; min-max: 3.9–54.7) and 9.6 μg/L (IQR: 11.1; min-max: 0.0–28.2), respectively (*p* = 0.012). Given the difference in hepcidin values depending on the occurrence or absence of hemolysis, hepcidin analyses were only performed with the nonhemolyzed samples.

Table 3 shows the results of the cord blood iron status parameters. No single woman had a C-reactive protein (CRP) value of >5mg/L in their cord blood sample (*N* = 73). Among the three women suffering from preeclampsia as pregnancy complication and delivered at 39w6d, 37w6d, and 38w, the corresponding hepcidin levels in their (nonhemolyzed) cord blood samples were 5.3, 12.0, and 37.1 μg/L, respectively, showing large interindividual variability.

Table 4 provides a detailed overview of the results of the correlation tests assessing a potential relationship between cord blood hepcidin and the continuous variables related to cord blood iron status and maternal pregestational BMI. Overall, a moderate positive relationship was found between cord blood hepcidin and ferritin (r = 0.493). Furthermore, a moderate negative relationship was observed between cord blood hepcidin and hemoglobin (r = −0.342). No other strong/moderate correlations were found with cord blood hepcidin.

Table 5 provides a detailed overview of the results of the association tests assessing a potential relationship between cord blood hepcidin and pregnancy/neonatal outcomes. The only association with cord blood hepcidin was found for the variable mode of delivery (*p* = 0.01), with higher hepcidin levels for vaginal deliveries compared to elective caesarean sections. Although no other associations were identified, the absolute difference in cord blood hepcidin levels with respect to prematurity is notable (8.7 µg/L), with premature neonates (born < 37 weeks) showing lower hepcidin levels.

Moreover, only one neonate had a birth weight of < 2500 g. As a result, no bivariate analysis was performed for the variable ‘low birth weight’. In the (hemolyzed) cord blood sample of this single neonate, an absolute hepcidin value of 7.25 µg/L was observed, which is much lower than the median hepcidin value in the overall cohort (i.e., 17.6 µg/L).

## 4. Discussion

This cross-sectional, observational study performed at a tertiary hospital in Belgium and using a validated hepcidin analysis method based on weak cation exchange chromatography and time-of-flight mass spectrometry, providing results similar to a standardized assay [24,27,28], aimed (1) to provide observational data on cord blood hepcidin levels in a Belgian cohort and (2) to determine variables associated with cord blood hepcidin levels.

Overall, 61 nonhemolyzed cord blood samples were collected and batch-analyzed. In total, a median hepcidin value of 17.6 µg/L was found, along with substantial variability in hepcidin levels among women (min-max: 3.9–54.7 µg/L). The observed median value is somewhat in line with the results of a previous study using the same method of analysis measuring hepcidin levels in cord blood of mothers with or without placental malaria infection and/or maternal anemia [30]. However, additional comparisons with hepcidin levels observed in other studies is difficult, given that many other studies used different (immunoassay or mass spectrometry) techniques and/or calibrators [13,15,17,19,31,32].

Moreover, in our cohort, we found a potential relationship between cord blood hepcidin and cord blood ferritin and hemoglobin, and mode of delivery. First, for cord blood ferritin, a moderate positive correlation was observed, in line with previous studies pointing at a relationship between cord blood hepcidin and cord blood iron status [12,13,14,15,16,17,18,19,20]. During pregnancy, maternal hepcidin concentrations also correlate with indicators of maternal iron status [5], which is also the case for healthy adult individuals [33]. Second, a negative correlation was found between hepcidin and hemoglobin levels in cord blood, similar to previous research [15]. Third, an association with mode of delivery was observed, with higher cord blood hepcidin levels for vaginal deliveries compared to elective caesarean sections, as shown earlier [14]. Finally, the observed trend towards higher cord blood hepcidin levels among term versus preterm neonates has also been previously shown [8,14].

Our study has some strengths. To our knowledge, this was the first study measuring hepcidin values in cord blood samples of pregnant women living in Belgium. Second, we used a validated and internationally accepted hepcidin analysis method [24], providing values similar to those obtained from a standardized assay. This enables the comparison with the findings of validated hepcidin assays used elsewhere, provided that they are standardized by using the same second reference material for calibration [27,28]. This will eventually reduce confusion in this field and ultimately allow for a global comparison of hepcidin values measured in cord blood. To achieve this, we collaborated with lab experts of the Radboud University Medical Center, who have extensive knowledge of and experience in hepcidin analysis, standardization, and interpretation of the results. International collaboration should always be pursued in this area, as it not only enables the sharing of expertise but also facilitates the application of fully validated analysis methods. Such approach may accelerate the acquisition of knowledge and insight into the actual clinical value of cord blood hepcidin. Third, we explored the potential relationship of cord blood hepcidin with maternal variables and pregnancy/neonatal outcomes that have previously been shown to be associated, despite conflicting results. So, we aimed to contribute to the replication of previous findings instead of testing numerous variables and running a (higher) risk of accidental findings (i.e., type I errors).

Some limitations should also be addressed. First, we acknowledge that the total number of 61 samples remains rather limited, which is partially explained/exacerbated by hemolysis of some samples. Due to the limited sample size, it cannot be excluded that for some variables, no significant results could be found (i.e., type II error). The limited sample size further avoided performing regression analyses and adjusting for potential confounders. Hence, we could not draw firm conclusions on potential relationships between cord blood hepcidin and other variables, and therefore consider our findings mainly explorative. A sample size or power calculation was not performed. Second, no maternal serum hepcidin levels in pregnancy and at the time of delivery were measured due to logistic and financial restraints. Future studies should further investigate the relative contribution of maternal/fetal hepcidin to placental iron transport [5,34]. Third, in our cohort, only few women were affected by preeclampsia or other pregnancy complications, hindering us to explore their relationship with cord blood hepcidin. Fourth, no (reliable) data were available on maternal iron status and the use of iron-containing medicines and/or supplements at the time of delivery, nor on ethnicity, although most—if not all—participants were probably Caucasian. Finally, we assumed that hemolysis of the samples occurred completely at random. Still, the finding that hepcidin levels were lower in hemolyzed samples could not be explained based on our relatively small cohort and requires further investigation.

Considering all the strengths and limitations of our work, our observational data and explorative analyses could hopefully contribute, to some extent, to establishing reference values of cord blood hepcidin [14] and to a better understanding of the potential of cord blood hepcidin for clinical and research purposes. Nevertheless, as various international round-robin tests performed by the Radboud group showed substantial differences in absolute levels measured by different assays—which decreased after standardization with their reference material [22,27,28,32,35]—specific attention should be paid to the harmonization and standardization of different hepcidin analysis methods.

## 5. Conclusions

In this observational study, hepcidin concentrations were measured in 61 nonhemolyzed cord blood samples using a validated weak cation exchange chromatography and time-of-flight mass spectrometry analysis method. Overall, a median hepcidin value of 17.6 µg/L was found, with a substantial variability in hepcidin levels among women. Moreover, a moderate positive and negative correlation was observed for cord blood hepcidin and cord blood ferritin and hemoglobin. A third potential association was identified for mode of delivery, with higher cord blood hepcidin levels for vaginal deliveries compared to elective caesarean sections. Although this exploratory study provided the first Belgian data on cord blood hepcidin levels, given its relatively limited sample size, larger studies collecting sufficient data on potential confounders are needed to provide more evidence on the actual value and benefit of cord blood hepcidin measurements for clinical and research purposes.

## Figures and Tables

**Table 1:** Overview of study participants’ baseline characteristics and pregnancy outcomes, according to cord blood hepcidin levels of the nonhemolyzed samples.

Variable	% (*n*)	Cord Blood Hepcidin Level
Maternal and pregnancy-related variables		
**Gravidity (*N* = 72)**		
Primigravida Multigravida	37.5 (27) 62.5 (45)	17.9 (19.8) 17.6 (19.0)
**Parity (*N* = 72)**		
Nullipara Primi- or multipara	61.1 (44) 38.9 (28)	18.0 (18.0) 15.5 (15.8)
**Pregestational body mass index (*N* = 70)**		
<25 kg/m^2^ ≥ 25 kg/m^2^	67.1 (47) 32.9 (23)	15.6 (19.1) 19.4 (14.3)
**Onset of pregnancy (*N* = 72)**		
Spontaneous Assisted	87.5 (63) 12.5 (9)	18.0 (15.2) 9.1 (14.4)
**Singleton or multiple pregnancy (*N* = 72)**		
Single Multiple	97.2 (70) 2.8 (2)	17.0 (17.9) 24.1 (/)
Pregnancy outcomes		
**Onset of labor (*N* = 71)**		
Spontaneous Induction of labor None (elective caesarian section)	50.7 (36) 33.8 (24) 15.5 (11)	18.4 (18.8) 18.0 (15.1) 7.3 (9.8)
**Mode of delivery (*N* = 72)**		
Vaginal Elective caesarean section Secondary caesarean section	79.2 (57) 15.3 (11) 5.6 (4)	18.4 (16.5) 7.3 (9.8) 17.9 (/)
**Gestational age at delivery (*N* = 72)**		
<34 weeks 34–37 weeks ≥37 weeks	0.0 (0) 8.3 (6) 91.7 (66)	/ 9.2 (19.7) 17.9 (16.2)

The results are shown as % (*n*). Hepcidin levels are shown as median (interquartile range, IQR) and are expressed in μg/L.

**Table 2:** Overview of the neonatal outcomes (*N* = 74) *.

Variable	% (*n*)
Prematurity ^1^	10.8 (8)
Low birth weight ^2^	1.4 (1)
Small for gestational age (SGA) ^3^	8.2 (6)
Large for gestational age (LGA) ^3^	12.3 (9)

The results are shown as % (*n*). * For the variables low birth weight, small for gestational age and large for gestational age, there was one missing value. ^1^ Prematurity was defined as being born <37 weeks gestational age. ^2^ Low birth weight was defined as a birth weight < 2500 g. ^3^ The results for small for gestational age (SGA; < 10th birth percentile) and large for gestational age (LGA; > 90th birth percentile) were calculated based on the customized criteria of the Study Centre on Perinatal Epidemiology (SPE) in Belgium, thereby accounting for gestational age, fetal sex, parity, and single/multiple pregnancies [29].

**Table 3:** Overview of the iron status parameters in cord blood ^1^.

Variable	*N*	Median (IQR)
Hepcidin (μg/L)	61	17.6 (18.1)
Hemoglobin (g/dL) ^2^	47	15.4 (1.8)
Ferritin (μg/L)	61	218 (179)
Iron (μg/dL)	61	152 (45)
Transferrin (g/L)	61	1.77 (0.35)
Transferrin saturation (%)	61	61 (21)

The results are shown as median (and interquartile range, IQR). The units for each variable are shown between brackets. ^1^ Only nonhemolyzed cord blood samples were considered for the analysis. ^2^ Some missing data exist for the variable hemoglobin.

**Table 4:** Relationship between cord blood hepcidin and (1) iron status and (2) pregestational BMI.

Variable	r ^1^	*p*-Value
Cord blood iron status parameters		
**Hemoglobin**	−0.342	0.02
**Ferritin**	0.493	<0.001
**Iron**	0.118	0.36
**Transferrin**	−0.098	0.45
**Transferrin saturation**	0.118	0.37
Maternal variable		
**Pregestational BMI** ^2^	0.241	0.07

^1^ The results were calculated using the Spearman correlation test and are reported using the correlation coefficient r and *p*-value. ^2^ BMI = body-mass index.

**Table 5:** Relationship between cord blood hepcidin and pregnancy/neonatal outcomes.

Variable	Median (IQR)	*p*-Value ^1^
Pregnancy outcomes		
**Onset of labor** Spontaneous Not spontaneous	18.4 (18.8) 15.8 (16.9)	0.23
**Mode of delivery ^2^** Vaginal Elective caesarean section	18.4 (16.5) 7.3 (9.8)	0.01
Neonatal outcomes		
**Prematurity**^3^ Yes No	9.2 (19.7) 17.9 (16.2)	0.21
**Small for gestational age (SGA)** ^4^ Yes No	25.9 (21.5) 17.6 (18.4)	0.19
**Large for gestational age (LGA)** ^4^ Yes No	19.8 (18.7) 17.6 (17.9)	0.89

^1^ The results were calculated using Mann–Whitney U tests and are reported using median (and interquartile range, IQR) and *p*-values. ^2^ The four women with a secondary caesarean section were not considered in order to not obscure the findings. ^3^ Prematurity was defined as being born < 37 weeks gestational age. ^4^ The results for small for gestational age (SGA; < 10th birth percentile) and large for gestational age (LGA; > 90th birth percentile) were calculated based on the customized criteria of the Study Centre on Perinatal Epidemiology in Belgium, thereby accounting for gestational age, fetal sex, parity, and single/multiple pregnancies [29].

## Data Availability

Not applicable.
